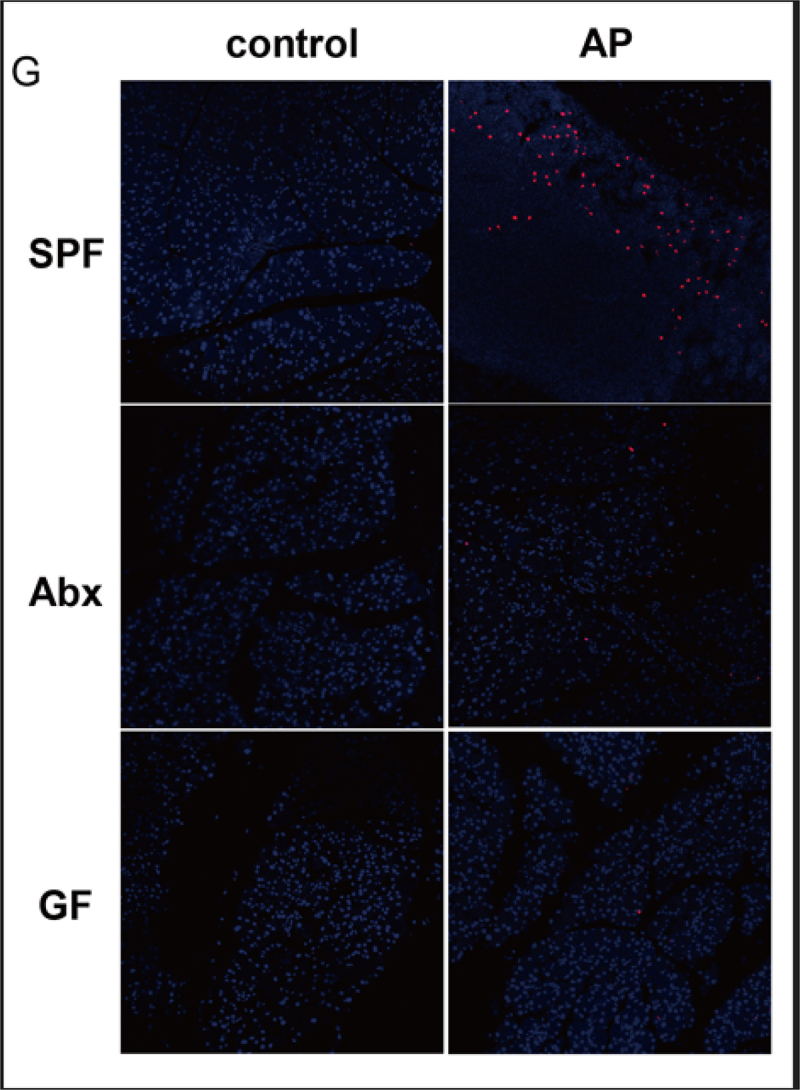# Correction

**DOI:** 10.1080/19490976.2024.2409026

**Published:** 2024-10-01

**Article title**: The interplay between the gut microbiota and NLRP3 activation affects the severity of acute pancreatitis in mice

**Authors**: Li, X., He, C., Li, N., Ding, L., Chen, H., Wan, J., Yang, X., Xia, L., He, W., Xiong, H., Shu, X., Zhu, Y., and Lu, N.

**Journal**: *KGMI: Gut Microbes*

**DOI**: https://doi.org/10.1080/19490976.2020.1770042

The article was originally published with the incorrect Figure 3G.

The figure 3G provided below have been included in the original article, and it has been republished accordingly.

**Figure:**